# Parity and gastric cancer risk: a systematic review and dose-response meta-analysis of prospective cohort studies

**DOI:** 10.1038/srep18766

**Published:** 2016-01-04

**Authors:** Jing Chen, Ting-Ting Gong, Qi-Jun Wu

**Affiliations:** 1 Department of Obstetrics and Gynecology, Shengjing Hospital of China Medical University, Shenyang, China; 2 Department of Clinical Epidemiology, Shengjing Hospital of China Medical University, Shenyang, China

## Abstract

We performed this meta-analysis of epidemiological studies to comprehensively assess the association between parity and gastric cancer risk, because previous studies have shown conflicting results regarding this topic. Relevant prospective studies were identified by searching the following databases: PubMed, EMBASE, and Web of Science, and random-effects models were used to estimate summary relative risks (SRRs) and 95% confidence intervals (CIs). Our search yielded 10 prospective cohort studies involving a total of 6624 gastric cancer cases and 5,559,695 non-cases. The SRRs for ever parity *vs.* nulliparous and highest *vs.* lowest parity number were 0.96 (95%CI = 0.87–1.05, *I*^2^ = 0%) and 1.03 (95%CI = 0.94–1.13, *I*^2^ = 0%), respectively. Additionally, the SRR for an increment of one live birth was 1.00 (95%CI = 0.97–1.03, *I*^2^ = 18.6%). These non-significant associations were observed in all subgroups as stratified by the number of gastric cases, follow-up years, geographic location, menopausal status, anatomic subsite of gastric cancer, and adjustment for potential confounders, as well as in sensitivity analyses. Our meta-analysis found no significant association between parity and gastric cancer risk. However, further studies should be conducted to validate our findings and could provide more detailed results by stratifying their findings by Lauren’s subtype, histology, and anatomic site, as well as fully adjusting for potential confounding factors.

In 2012, gastric cancer was the fourth most common type of cancer and the third leading cause of cancer mortality among both men and women worldwide^1^. The age standardized incidence and mortality rate for gastric cancer were 17.4 and 12.8 per 100,000 individuals, respectively among both men and women^2^. Although the incidence of gastric cancer varies widely among different countries, its incidence is generally 2-fold higher in men compared to women^1^. Because this discrepancy in occurrence cannot be totally attributed to environmental factors (e.g., cigarette smoking and fruit and vegetable intake) or *Helicobacter pylori* (*H. pylori*) infection^3^, some investigators have proposed that sex hormonal factors (e.g., parity, hormone replacement therapy, and age at menarche), and especially estrogen levels, might play etiological roles in gastric cancer^4^,^5^,^6^,^7^.

Pregnant women have markedly elevated serum levels of certain hormones, including estrogens^8^. Results of our previous studies suggested that parity was significantly associated with the risk for kidney cancer, but not the risk for colorectal or pancreatic cancer^9^,^10^,^11^. Although several studies have suggested that parity might be associated with gastric cancer risk through its effects on steroid hormone metabolism and related pathways^4^,^12^,^13^,^14^, the published epidemiological evidence for this hypothesis is inconclusive; possible due to the limited number of gastric cancer cases investigated^8^,^15^,^16^,^17^,^18^,^19^,^20^,^21^,^22^,^23^. Some investigators have argued that pregnancy-related hormones may protect against gastric cancer, whereas others have argued that they have a stimulatory effect or no effect at all. Furthermore, the most recent meta-analysis (conducted in 2011) of 12 observational studies with mixed study designs and quality found limited evidence for the aforementioned associations, and a high degree of heterogeneity in the study results^24^. Notably, that previous meta-analysis did not include a dose-response analysis to evaluate associations regardless of their category. Hence, we conducted a systematic review and meta-analysis which included the most prospective studies to comprehensively and quantitatively evaluate the relationship between parity and the risk for developing gastric cancer.

## Results

### Search results, study characteristics, and quality assessment

**Figure 1:**
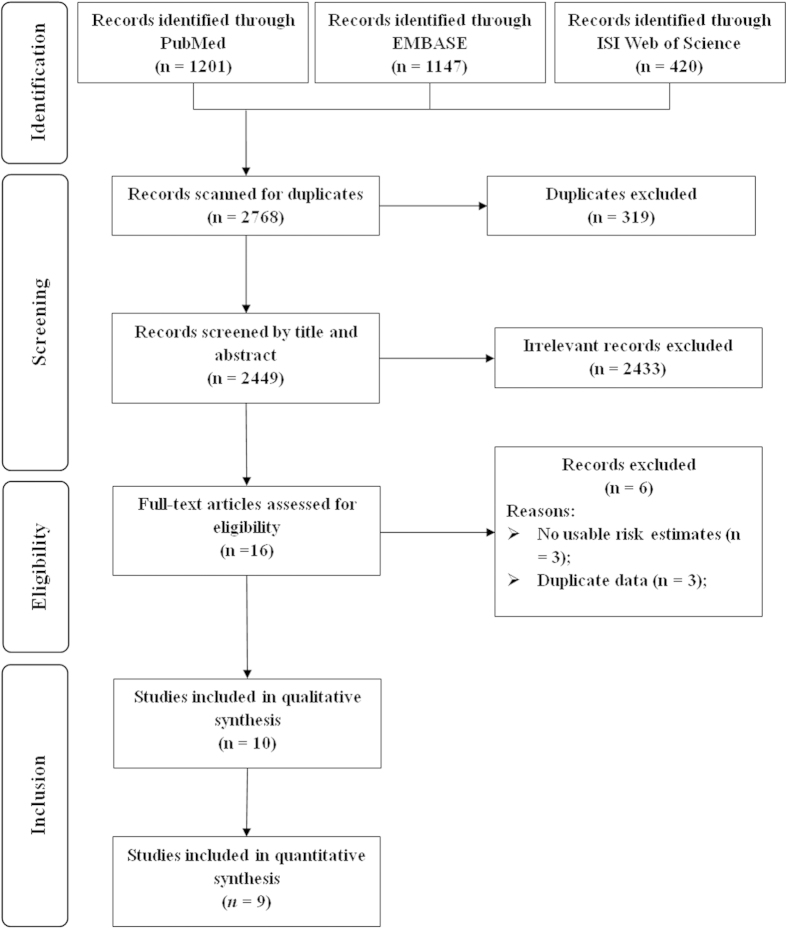
Selection of studies for inclusion in meta-analysis.

The detailed procedures used for searching and screening articles are outlined in Fig. 1. In brief, our search strategy retrieved 2449 unique reports after excluding the duplications: 1201 from PubMed, 1147 from EMBASE, and 420 from Web of Science. Of these, 2433 reports were excluded after the first screening based on their abstracts or titles, leaving 16 reports eligible for full-text review. Among these 16 reports, 6 were excluded due to i) a lack of usable risk estimates or 95% CIs or ii) study population duplication. Finally, a total of 10 prospective studies were included in the present meta-analysis^8^,^15^,^16^,^17^,^18^,^19^,^20^,^21^,^22^,^23^.

Characteristics of the 10 selected studies are shown in Table 1. These studies were published between 2000 and 2012 and involved a total of 6624 gastric cancer cases and 5,559,695 non-cases. Five of the 10 studies were conducted in Europe, four were conducted in Asia, and one was conducted in the United States. A majority of risk measures were adjusted for or stratified by age (7 studies); however, fewer studies were adjusted for cigarette smoking (5 studies), body mass index (4 studies), alcohol consumption (3 studies), and socioeconomic status (5 studies).

Information regarding the quality assessment of studies is provided in Supplementary Table S1. Briefly, when controlling for an important factor or an additional factor category, five of the included studies^8^,^16^,^19^,^21^,^23^ were not assigned two scores because results of their primary analyses were not adjusted for potential confounders. When testing for whether a study’s follow-up time was long enough for outcomes to occur, five of the included studies^8^,^16^,^19^,^21^,^23^ received a score because their mean follow-up period was >10 years.

### Ever parity versus nulliparous

**Figure 2:**
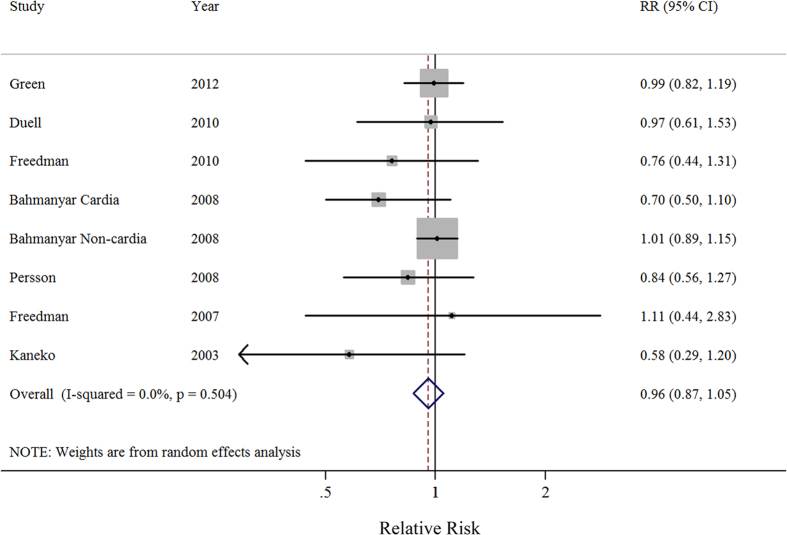
Forest plot (random-effects model) of ever parity and gastric cancer risk. The squares indicate study-specific relative risks (size of the square reflects the study specific statistical weight); the horizontal lines indicate 95% CIs; and the diamond indicates the summary relative risk estimate with its 95% CI.

Seven prospective studies investigated the relationship between ever parity and the risk of gastric cancer. A comparison of ever parity *vs.* nulliparous yielded a SRR of 0.96 (95%CI = 0.87–1.05), without heterogeneity (*I*^2^ = 0%) (Fig. 2). Both a visual inspection of a funnel plot and Egger’s test (*P* = 0.159) failed to show evidence of publication bias, and similar non-significant results were observed in all stratified analyses performed based on study characteristics. Additionally, the results of a meta-regression showed no evidence of significant heterogeneity between subgroup analyses (Table 2).

### High parity number versus low parity number

**Figure 3:**
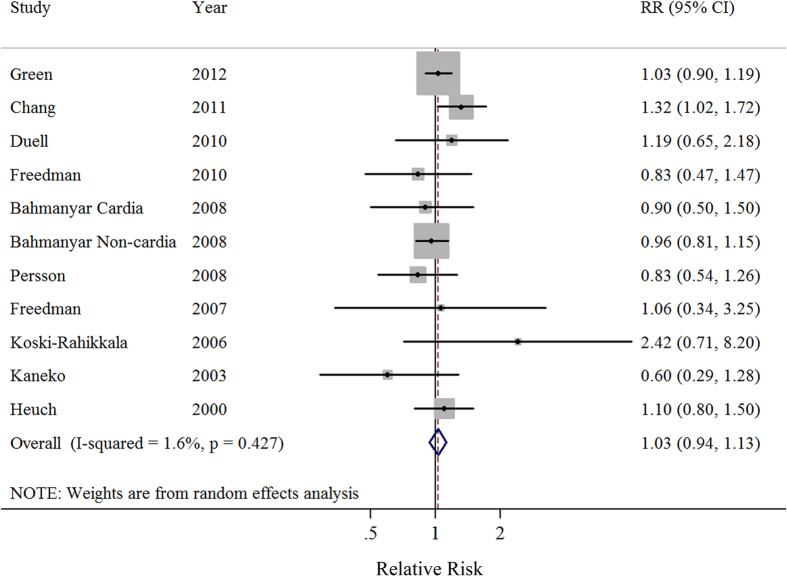
Forest plot (random-effects model) of parity number (highest *vs.* lowest) and gastric cancer risk. The squares indicate study-specific relative risks (size of the square reflects the study specific statistical weight); the horizontal lines indicate 95% CIs; and the diamond indicates the summary relative risk estimate with its 95% CI.

Ten prospective studies investigated the relationship between parity number and the risk for gastric cancer. A comparison of the highest *vs.* the lowest parity number yielded a SRR of 1.03 (95%CI = 0.94–1.13), without heterogeneity (*I*^2^ = 1.6%) (Fig. 3). Similar non-significant results were also observed in all of the stratified analyses performed based on study characteristics. Additionally, the results of a meta-regression showed no evidence of significant heterogeneity between these subgroup analyses (Table 3).

### Dose-response analysis

**Figure 4:**
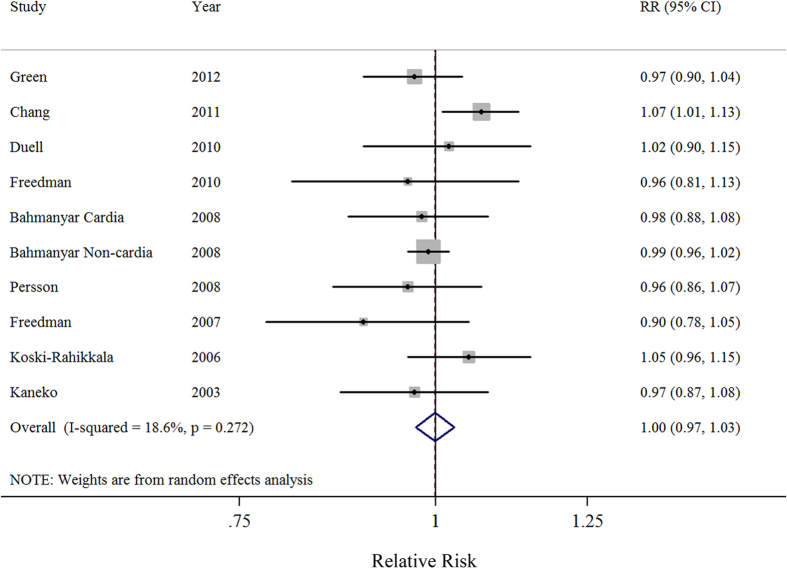
Forest plot (random-effects model) of parity number (per 1 live birth) and gastric cancer risk. The squares indicate study-specific relative risks (size of the square reflects the study specific statistical weight); the horizontal lines indicate 95% CIs; and the diamond indicates the summary relative risk estimate with its 95% CI.

Nine prospective studies were included in the dose-response analysis. Results showed the summary relative risk per live birth was 1.00 (95%CI = 0.97–1.03), with low heterogeneity (*I*^2^ = 18.6%) (Fig. 4). There was no evidence for a nonlinear association between the parity number and gastric cancer risk (*P* for nonlinearity = 0.093) (Supplementary Figure S1). Similar non-significant results were also observed in all of the stratified analyses performed based on study characteristics. Additionally, the results of a meta-regression showed no evidence of significant heterogeneity between these subgroup analyses (Table 3).

### Sensitivity analysis

Our sensitivity analysis of ever parity *vs.* never parity performed by excluding one study at a time showed that the SRR for gastric cancer ranged from 0.93 (95%CI = 0.80–1.08; *I*^2^ = 0%) when Bahmanyar *et al.*^8^ was excluded, to 0.96 (95%CI = 0.88–1.06; *I*^2^ = 0%), when Persson *et al.*^19^ was excluded. Similarly, in the sensitivity analysis of high parity number *vs.* low parity number, the SRR for gastric cancer ranged from 0.99 (95%CI = 0.91–1.09; *I*^2^ = 0%) when Chang *et al.*^16^ was excluded, to 1.06 (95%CI = 0.93–1.21; *I*^2^ = 11.7%) when Bahmanyar *et al.*^8^ was excluded. Additionally, in the sensitivity analysis of dose response, the SRR for gastric cancer ranged from 0.99 (95%CI = 0.96–1.01; *I*^2^ = 0%) when Chang *et al.*^16^ was excluded, to 1.00 (95%CI = 0.97–1.04; *I*^2^ = 22.7%) when Green *et al.*^15^ was excluded. When the analysis of highest *vs.* lowest parity number was restricted to studies included in the dose-response analysis of parity number, the SRR was 1.02 (95%CI = 0.92–1.14; *I*^2^ = 9.7%), which is similar to that in the original analysis. Furthermore, we excluded three studies which reported their risk estimates based on mortality resulting from gastric cancer. However, even after excluding those studies, the results remained statistically non-significant (data not shown).

## Discussion

The findings from this meta-analysis of prospective studies only marginally suggested that parity might help protect against gastric cancer. To the best of our knowledge, this is the most comprehensive and quantitative assessment of the aforementioned association conducted using high quality evidence. Additionally, these non-significant results were consistent with those in subgroup analyses stratified by study characteristics and adjustments for potential confounders.

Increasing evidence has suggested that there are differences in risk factors associated with developing different Lauren’s subtypes of gastric cancer (intestinal *vs.* diffuse) and gastric cancers located at different anatomic sites (cardia *vs.* non-cardia)^24^,^25^. Furthermore, the biology and etiology is varied by histological type of this disease. However, only one and three of the included studies reported risk estimates stratified by Lauren’s subtype^19^ and the anatomic site of gastric cancer^8^,^15^,^18^ respectively. Notably, only three studies^15^,^17^,^18^ provided the aforementioned association on the basis of the adenocarcinomas of gastric cancer. Therefore, our meta-analysis was insufficient to draw conclusions concerning the risk for developing gastric cancer in relation to parity or based on cancer subtype, histology and location.

Previous studies demonstrated that hormones might help protect against gastric cancer during a woman’s fertile years, but their effect diminishes after menopause^26^,^27^. The results of several studies support this hypothesis, because they showed that rates of gastric cancer increase more slowly in women compared to men until the age of 60 years; after which, gastric cancer rates in women increase rapidly and become more similar to those in men^20^,^26^. However, until recently, similar to the previous discussion of anatomical subtypes of gastric cancer, only a limited number of prospective studies have included a subgroup analysis stratified by menopausal status. This is because the majority of studies had insufficient numbers of gastric cancers to allow a comparison of risks in pre- and postmenopausal women. Although the results of our meta-regression showed no difference regarding the gastric cancer risk in pre- and postmenopausal women, only three studies provided a risk estimate stratified by menopausal status^8^,^19^,^22^, and thus additional studies are warranted to examine the association between menopausal status and gastric cancer risk after eliminating the possibility of chance findings.

Although our present meta-analysis found limited evidence for a relationship between parity and the risk of developing gastric cancer, several potential biological mechanisms might cause such an association. Results from previous studies have suggested that sex hormones, including estrogens, might inhibit the development and progression of gastric cancer by increasing an individual’s resistance to inflammation and inhibiting production of gastric acid and gastrin. For example, estrogens are known to regulate numerous physiological processes primarily by binding to estrogen receptors, which are potent regulators of gene transcription^47^. As estrogen receptors are present in gastric epithelial tissue^28^,^29^, and have been shown to inhibit inflammation^30^,^31^,^32^, it is biologically plausible that estrogens might protect against gastric cancer. Furthermore, estrogens are known to directly affect production gastric acid and progesterone, and indirectly affect gastrin production via somatostatin^33^,^34^.

Our study has several strengths that should be mentioned. To the best of our knowledge, this is the most comprehensive and quantitative assessment of the relationship between parity and the risk for developing gastric cancer. Our current meta-analysis included results from 10 prospective studies which enrolled a total of 6624 gastric cancer cases and 5,559,695 non-cases, and thus had sufficient statistical power to detect an association between parity and gastric cancer. Furthermore, we divided parity into several categories (i.e., ever parity *vs.* nulliparous; high parity number *vs.* low parity number) instead of merely presenting results representing a summary of all different categories of parity (Table 2 and Table 3). Notably, the results of our numerous subgroup and sensitivity analyses were consistent with the main findings and without heterogeneity, albeit they revealed no statistically significant differences.

This study also has several limitations that should be mentioned. First, this meta-analysis was based on the observational designs of the included prospective studies, and confounding is a concern in all observational studies. All of the included studies except one^21^ reported their risk estimates based on multivariable models. However, several studies made adjustments for important potential confounders such as cigarette smoking, body mass index, alcohol consumption, and socioeconomic status in their primary analyses (Table 1). Thus it cannot be ruled out that the observed association between parity and gastric cancer risk might be explained by residual confounding. Furthermore, it is possible that unmeasured or unidentified risk factors may have affected the results even after controlling for potential confounders. Notably, all of the included studies lacked information concerning *H. pylori*, which is an important risk factor of gastric cancer. The lack of this information severely limited our ability to adjust for or conduct a proper subgroup analysis stratified by *H. pylori* infection. Several studies have indicated that an increased susceptibility to *H. pylori* infection during pregnancy might be related to an increased risk of gastric cancer^35^,^36^. In contrast, Freedman *et al.*^18^ mentioned that *H. pylori* infection did not confound an observed association between menstrual and reproductive factors and gastric cancer risk. Therefore, future studies are needed to analyze such important confounders and adjust for or stratify them along with other risk factors to rule out possible residual confounding. Second, in contrast to the previous register-based analyses, the parity information used in our analysis was collected from self-reports included in several prospective studies, and this might have introduced errors due to misclassification. Although parity information is generally well-reported and highly reliable when compared with reports concerning other reproductive factors, a non-differential misclassification of parity may have slightly shifted risk estimates towards the null value. However, our subgroup analyses stratified by the aforementioned variable produced results similar to the main findings, and showed no significant differences (data not shown). Third, although the outcomes, the incidence of gastric cancer, were identified via record linkage to the cancer registries, the criteria and procedure of diagnosis of this disease might be slightly different among these studies. For example, three included studies^15^,^17^,^22^ codes gastric cancer according to the 10th revision of the WHO International Classification of Diseases (ICD-10). Two included studies^16^,^20^ codes this disease by the ICD-9. By comparison, Bahmanyar *et al.*^8^ and Heuch *et al.*^23^ both used ICD-7 in their studies. The site and histology of gastric cancer cases were coded using the International Classification of Diseases for Oncology, Third Edition in study of Persson *et al.*^19^. Last, although levels of endogenous hormone obviously change throughout pregnancy, parity might be an imperfect marker of overall exposure to sex hormones, because when compared to the life-span of a woman, the exposure period is too short-lived or inadequately timed to have an appreciable effect gastric carcinogenesis^8^. Therefore, further analyses which include adjustments or stratification for other menstrual and reproductive factors need to be conducted in the future.

In summary, our meta-analysis of prospective studies found no association between parity and the risk for developing gastric cancer. The reliability of our finding is increased by the fact that evidence from prospective studies is generally considered to be more reliable than evidence from retrospective studies. However, with regards to public health recommendations, our findings are complicated by the fact that parity may have both beneficial and adverse effects with regards to other diseases. Further studies of parity in relation to other cancers as well as overall cancer risk and mortality are needed to better assess the risk-benefit of exposure to pregnancy-related hormones.

## Material and Methods

### Search strategy

Two independent investigators (T-TG and Q-JW) systematically searched PubMed (MEDLINE), EMBASE, and Web of Science for relevant prospective studies published starting from the time of each database’s inception to May 30, 2015. The following keywords were used for searching: (parity OR pregnancy OR livebirth OR reproductive OR reproduction) AND (stomach OR gastric) AND (cancer OR tumor OR carcinoma OR neoplasm). This search strategy was similar to that used in our previous studies^9^,^10^,^11^,^25^,^37^. Guidelines in Preferred Reporting Items for Systematic Reviews and Meta-Analyses (PRISMA) were followed when planning, conducting, and reporting this meta-analysis^38^.

### Study selection and exclusion

Two independent investigators (JC and T-TG) performed the study selection and exclusion procedures. Studies included in this analysis were required to satisfy the following criteria: (i) utilized a prospective study design; (ii) evaluated the association between parity and the risk for developing gastric cancer; (iii) presented estimates of relative risk (RR) or hazard ratio (HR) with 95% confidence intervals (CIs) or data necessary to calculate those parameters. If several publications involved overlapping patients, the study with the most patients was included.

The following types of studies were excluded from our meta analysis: (i) randomized controlled trials, case-control studies, retrospective studies, reviews without original data, ecological studies, editorials, and case reports; (ii) studies reported with risk estimates that could not be summarized (e.g., studies reported without 95%CIs).

### Data abstraction and quality assessment

Data were extracted using a data extraction form and entered into a database by a single investigator (Q-JW). Next, an independent investigator (JC) checked the data, and all differences were resolved by a third investigator (T-TG). The following information was extracted for each included study: last name of the first author, publication year, geographic location of the study, number of cases/size of cohort, follow-up years, exposure and outcome assessment, categories of exposure, and study-specific adjusted estimates with their 95%CIs (including adjusted confounder information if applicable). If there were multiple estimates for the association, we used the estimate adjusted for the most appropriate confounding variables. This was similar to the method used in other studies^39^,^40^,^41^,^42^,^43^.

The Newcastle-Ottawa Scale (NOS)^25^,^39^,^40^,^41^,^42^,^43^,^44^,^45^ uses three quality parameters (selection, comparability, and exposure/outcome), and was used to assess the methodological quality of the studies included in our meta-analysis. We used the NOS instead of a scoring system that divides studies into categories of high or low quality, because quality scoring might not only hide important information by combining disparate study features into a single score, but also introduce an arbitrary subjective element into the analysis^46^,^47^,^48^.

### Statistical analysis

We used the counting method proposed by Hamling *et al.*^49^ to recalculate the relative risks in studies^15^,^17^,^18^,^19^,^20^,^22^ that did not provide results for ever parity *vs.* nulliparous, and studies^15^,^20^ that did not use the category with the lowest parity number as a reference. The results from one study^8^ that reported separate data for cardia and non-cardia gastric cancer were pooled with the results of other studies.

To examine associations between parity and gastric cancer risk, the summarized relative risks (SRRs) with their 95% CIs were estimated by summarizing the risk estimates of each study using random effect models that considered both within- and between-study variations^50^. Furthermore, we summarized the study-specific SRR for each one live birth increment in the parity number. A study-specific trend reflecting correlated log RRs across different categories of parity number was computed using the generalized least-squares trend estimation method developed by Greenland *et al.*^51^ and Orsini *et al.*^52^. Furthermore, a potential nonlinear dose-response relationship between parity number and gastric cancer risk was modeled by using restricted cubic splines with 3 knots at fixed percentiles (10%, 50%, and 90%) of the distribution of exposure. An overall *P* value was calculated by showed that these two regression coefficients simultaneously equaled zero. The *P* value for nonlinearity was calculated by showing that the coefficient of the second spline equaled zero. The details of this method for calculating *P* values have been previously described^53^,^54^.

The following information was required and used to conduct the dose-response meta-analysis: 1) the distribution of cases and non-cases, and the risk estimates with the variance estimates for at least three quantitative exposure categories; 2) the median or mean level of these exposures in each category (if reported by ranges, mean levels were calculated by averaging the lower and upper bound; if the lowest category was open ended, the lowest boundary was considered to be zero; if the highest category was open ended, the open-ended interval length was assumed to be the same as the adjacent interval)^37^. When using these techniques, nine studies^8^,^15^,^16^,^17^,^18^,^19^,^20^,^21^,^22^ met our specifications, and were included in the dose-response analysis of parity number and gastric cancer risk.

The *I*^2^ metric was used to evaluate between-study heterogeneity. Values for *I*^2^ range between 0% and 100%, and represent the ratio of between-study variance divided by the sum of the within-study and between-study variances^55^. Pre-specified subgroup analyses were conducted based on the number of gastric cases (≥250 *vs.* <250), number of follow-up years (≥10 *vs.* <10), geographic location (North America, Europe or Asia), patient menopausal status (pre-menopausal *vs.* post-menopausal), anatomic subsite of the gastric cancer (cardia *vs.* non-cardia), and adjustments made for potential confounders including body mass index, cigarette smoking, alcohol consumption, and socioeconomic status. Heterogeneity between subgroups was evaluated by meta-regression^25^,^39^,^41^,^56^,^57^. Egger’s regression asymmetry test^43^ was used to test for small study biases, such as publication bias, that can reflect genuine heterogeneity, chance, or other reasons for differences between small and large studies. A *P*-value of 0.05 was used to determine whether significant publication bias existed. Furthermore, sensitivity analyses were conducted by deleting each study in turn to examine the influence of individual data on the overall estimate. All statistical analyses were performed using Stata (version 12; StataCorp, College Station, TX, USA).

## Additional Information

**How to cite this article**: Chen, J. *et al.* Parity and gastric cancer risk: a systematic review and dose-response meta-analysis of prospective cohort studies. *Sci. Rep.* **6**, 18766; doi: 10.1038/srep18766 (2016).

## Supplementary Material

Supplementary Information

## Figures and Tables

**Table 1:** Characteristics of included prospective studies of parity and gastric cancer risk.

First author, publication year (reference), Country	Cases/subject (age), duration of follow up	Exposure categories (exposure/case assessment)	RR (95% CI)	Matched/Adjusted factors
Green *et al.*^15^^,^† 2012, United Kingdom	1194/1,319,409 (50–64y), 9.1y	Parity: Ever parous vs. Nulliparous Parity: :≥3 vs. 2 (Self-questionnaire/Cancer registry)	0.99 (0.82–1.19) 1.03 (0.90–1.19)	Stratified by age, region and socioeconomic status and adjusted for BMI, smoking, alcohol, strenuous exercise, use of OC and for all other reproductive factors and for use of hormone therapy for the menopause
Chang *et al.*^16^^,^† ‡ 2011, Taiwan, China	1090/1,292,462 (40–69y), 31y	Parity: :≥3 vs. Nulliparous (Registration form/Cancer registry)	1.32 (1.02–1.72)	Age, marital status, years of schooling, and birth place
Duell *et al.*^17^, 2010, Europe	181/335,216 (35–70y), 8.7y	Parity: Ever parous vs. Nulliparous Parity: :≥4 vs. Nulliparous (Self-questionnaire/Cancer registry)	0.97 (0.61–1.53) 1.19 (0.65–2.18)	Age, center, smoking status, education, BMI, and calorie-adjusted vegetable, fruit, red meat, and processed meat intakes
Freedman *et al.*^18^, 2010, USA	97/201,506 (50–71y), 7.5y	Non-cardia gastric cancer Parity: Ever parous vs.Nulliparous Parity: :≥3 vs. Nulliparous (Self-questionnaire/Cancer registry)	0.76 (0.44–1.31) 0.83 (0.47–1.47)	Age, BMI, fruit and vegetable consumption, smoking use, alcohol intake, physical activity, and total energy intake
Bahmanyar *et al.*^8^, 2008, Sweden	2784/2,406,439 (≥30y), 34y	Cardia gastric cancer Parity: Ever parous vs. Nulliparous Parity: :≥4 vs. 1 Non-cardia gastric cancer Parity: Ever parous vs. Nulliparous Parity: :≥4 vs. 1 (Self-questionnaire/Cancer registry)	0.70 (0.50–1.10) 0.90 (0.50–1.50) 1.01 (0.89–1.15) 0.96 (0.81–1.15)	Occupational class, education level, and age at first birth Age, family history of gastric cancer, and study area
Persson *et al.*^19^^,^† 2008, Japan	368/44,453 (40–69y), 12.2y	Parity: Ever parous vs. Nulliparous Parity: :≥3 vs. Nulliparous (Self-questionnaire/Cancer registry)	0.84 (0.56–1.27) 0.83 (0.54–1.26)	
Freedman *et al.*^20^^,^† 2007, China	154/73,442 (40–70y), 5.7y	Parity: Ever parous vs. Nulliparous Parity: :≥4 vs. Nulliparous (Self-questionnaire/Cancer registry)	1.11 (0.44–2.82) 1.06 (0.34–3.25)	Age, BMI, education, income, cigarette smoking status, and smoking dose
Koski-Rahikkala *et al.*^21^^,^‡ ¶2006, Finland	28/12,002 (mean, 27.8y), 36y	Parity: :≥10 vs. 2–4 (Self-questionnaire/Cancer registry)	2.42 (0.71–8.20)	N/A
Kaneko *et al.*^22^^,^† ‡ 2003, Japan	156/120,000 (40–79y), 8.2y	Parity: Ever parous vs. Nulliparous Parity: :>3 vs. Nulliparous (Self-questionnaire/Cancer registry)	0.58 (0.29–1.20) 0.60 (0.29–1.28)	Smoking status, family history, past history of peptic ulcer, alcohol intake, educational background, number of rice bowls, and dietary consumption
Heuch *et al.* 23^,^† 2000, Norway	572/63,090 (32–74y), 29y	Parity: :≥5 vs. 1 (Self-questionnaire/Cancer registry)	1.10 (0.80–1.50)	Age, birth cohort, urban/rural residence and county

BMI: body mass windex; CI: confidence interval; N/A: not available; OC: oral contraceptive; RR: relative risk.

^†^Recalculate the RR by the method proposed by Hamling *et al.*^49^.

^‡^Using mortality data to calculate risk estimates.

^¶^Relative risks and w95% CIs were calculated from published data using EpiCalc 2000.

**Table 2:** Summary risk estimates of the association between ever parity and gastric cancer risk.

	No. of Study	SRR	95% CI	*I*^2^ (%)	*P*_h_†	*P*_h_‡
**Overall**	7	0.96	0.87–1.05	0	0.504	
**Subgroup analyses**						
**Number of cases**						0.383
≥250	3	0.96	0.85–1.08	15.1	0.317	
<250	4	0.84	0.62–1.13	0	0.594	
**Follow-up years**						0.974
≥10	2	0.90	0.87–1.05	42.4	0.176	
<10	5	0.94	0.81–1.11	0	0.593	
**Geographic location**						0.221
Europe	3	0.98	0.89–1.08	0.8	0.388	
Asia	3	0.80	0.58–1.12	0	0.517	
North America	1	0.76	0.44–1.31	N/A	N/A	
**Menopausal status**						0.962
Pre-menopausal	1	0.83	0.65–1.05	N/A	N/A	
Post-menopausal	3	0.83	0.61–1.13	66.7	0.029	
**Anatomic subsite**						0.279
Cardia	2	0.81	0.61–1.07	1.2	0.314	
Non-cardia	3	0.99	0.88–1.12	0	0.589	
**Adjustment for potential confounders and risk factors**						
**Age**						0.769
Yes	5	0.95	0.82–1.10	0	0.862	
No	2	0.82	0.59–1.15	60.0	0.082	
**Cigarette smoking**						0.974
Yes	5	0.95	0.81–1.11	0	0.593	
No	2	0.90	0.72–1.12	42.4	0.176	
**Body mass index**						0.617
Yes	4	0.97	0.82–1.14	0	0.827	
No	3	0.85	0.68–1.08	44.3	0.146	
**Alcohol drinking**						0.844
Yes	3	0.88	0.67–1.15	24.6	0.265	
No	4	0.97	0.86–1.08	0	0.468	
**Socioeconomic status**						0.340
Yes	5	0.97	0.87–1.07	3	0.397	
No	2	0.81	0.58–1.12	0	0.774	

CI, confidence interval; N/A, not available; SRR, summarized relative risk.

^†^*P*-value for heterogeneity within each subgroup.

^‡^*P*-value for heterogeneity between subgroups with meta-regression analysis.

**Table 3:** Summary risk estimates of the association between parity number and gastric cancer risk.

	Highest *vs.* lowest						Dose-response analysis (per 1 live birth)					
	No. of Study	SRR	95% CI	*I*^2^ (%)	*P*_h_†	*P*_h_‡	No. of Study	SRR	95% CI	*I*^2^ (%)	*P*_h_†	*P*_h_‡
**Overall**	10	1.03	0.94–1.13	1.6	0.427		9	1.00	0.97–1.03	18.6	0.272	
**Subgroup analyses**												
**Number of cases**						0.693						0.826
≥250	5	1.04	0.94–1.14	8.7	0.361		4	1.00	0.96–1.04	45.7	0.118	
<250	5	0.96	0.67–1.38	11.1	0.343		5	1.00	0.94–1.05	0	0.454	
**Follow-up years**						0.618						0.217
≥10	5	1.06	0.89–1.25	31.5	0.199		4	1.01	0.97–1.06	46.9	0.110	
<10	5	1.01	0.89–1.15	0	0.607		5	0.97	0.92–1.02	0	0.802	
**Geographic location**						0.976						0.968
Europe	5	1.02	0.92–1.13	0	0.690		4	0.99	0.97–1.02	0	0.707	
Asia	4	0.98	0.67–1.42	51.1	0.105		4	0.99	0.91–1.07	60.2	0.057	
North America	1	0.83	0.47–1.47	N/A	N/A		1	0.96	0.81–1.13	N/A	N/A	
**Menopausal status**						0.794						0.831
Pre-menopausal	1	0.80	0.46–1.40	N/A	N/A		1	0.93	0.77–1.12	N/A	N/A	
Post-menopausal	3	0.90	0.76–1.07	0	0.468		3	0.97	0.94–1.00	0	0.773	
**Anatomic subsite**						0.879						0.342
Cardia	2	1.04	0.80-1.34	0	0.566		2	0.93	0.82–1.05	49.4	0.160	
Non-cardia	3	1.01	0.85–1.20	13.2	0.316		3	0.99	0.96–1.02	0	0.876	
**Adjustment for potential confounders and risk factors**												
**Age**						0.276						0.939
Yes	7	1.06	0.96–1.18	0	0.538		6	0.99	0.94–1.05	43.1	0.118	
No	3	0.94	0.72–1.22	20.4	0.288		3	0.99	0.97–1.02	0	0.626	
**Cigarette smoking**						0.618						0.217
Yes	5	1.01	0.89–1.15	0	0.607		4	0.97	0.92–1.02	0	0.802	
No	5	1.06	0.89–1.25	31.5	0.199		5	1.01	0.97–1.06	46.9	0.110	
**Body mass index**						0.938						0.285
Yes	4	1.03	0.90–1.17	0	0.857		4	0.97	0.92–1.02	0	0.652	
No	6	1.03	0.86–1.23	36.1	0.153		5	1.01	0.97–1.05	37.2	0.159	
**Alcohol drinking**						0.475						0.323
Yes	3	0.96	0.77–1.20	16.7	0.301		3	0.97	0.92–1.03	0	0.993	
No	7	1.06	0.93–1.20	6.4	0.381		6	1.01	0.97–1.05	38.2	0.137	
**Socioeconomic status**						0.257						0.056
Yes	5	0.99	0.90–1.10	0	0.753		5	0.98	0.96–1.01	0	0.836	
No	5	1.10	0.87–1.38	33.7	0.196		4	1.03	0.98–1.09	24.6	0.264	

CI, confidence interval; N/A, not available; SRR, summarized relative risk.

^†^*P*-value for heterogeneity within each subgroup.

^‡^*P*-value for heterogeneity between subgroups with meta-regression analysis.
