# Supplementary material for: Parity and gastric cancer risk: a systematic review and dose-response meta-analysis of prospective cohort studies
Source: Sci Rep. 2016 Jan 4;6:18766. doi: 10.1038/srep18766 (PMC4698715; doi:10.1038/srep18766)
Supplement: Supplementary Information [file srep18766-s1.pdf]

# Parity and gastric cancer risk: a systematic review and dose-response meta-analysis of prospective cohort studies

Jing Chen, Ting-Ting Gong, Qi-Jun Wu

**Supplementary Table S1.** Methodological quality of prospective studies included in the meta-analysis*

| First author, (reference) publication year | Representativeness of the exposed cohort | Selection of the unexposed cohort | Ascertainment of exposure | Outcome of interest not present at start of study | Control for important factor or additional factor† | Assessment of outcome | Follow-up long enough for outcomes to occur ‡ | Adequacy of follow-up of cohorts § |
|---|---|---|---|---|---|---|---|---|
| Green, 2012 | ☆ | ☆ | ☆ | ☆ | ☆☆ | ☆ | — | ☆ |
| Chang, 2011 | ☆ | ☆ | ☆ | ☆ | — | ☆ | ☆ | ☆ |
| Duell, 2010 | ☆ | ☆ | ☆ | ☆ | ☆☆ | ☆ | — | ☆ |
| Freedman, 2010 | ☆ | ☆ | ☆ | ☆ | ☆☆ | ☆ | — | ☆ |
| Bahmanyar, 2008 | ☆ | ☆ | ☆ | ☆ | — | ☆ | ☆ | ☆ |
| Persson, 2008 | ☆ | ☆ | ☆ | ☆ | — | ☆ | ☆ | ☆ |
| Freedman, 2007 | ☆ | ☆ | ☆ | ☆ | ☆☆ | ☆ | — | ☆ |
| Koski-Rahikkala, 2006 | ☆ | ☆ | ☆ | ☆ | — | ☆ | ☆ | ☆ |
| Kaneko, 2003 | ☆ | ☆ | ☆ | ☆ | ☆☆ | ☆ | — | ☆ |
| Heuch, 2000 | ☆ | ☆ | ☆ | ☆ | — | ☆ | ☆ | ☆ |

* A study could be awarded a maximum of one star for each item except for the item Control for important factor or additional factor. The definition/explanation of each column of the Newcastle-Ottawa Scale is available from (http://www.ohri.ca/programs/clinical_epidemiology/oxford.asp.).

† A maximum of 2 stars could be awarded for this item. Studies that controlled for cigarette smoking received one star, whereas studies that controlled for other important confounders such as body mass index, alcohol drinking received an additional star.

‡ A cohort study with a follow-up time ≥10 y was assigned one star.

§ A cohort study with a follow-up rate ≥75% was assigned one star.

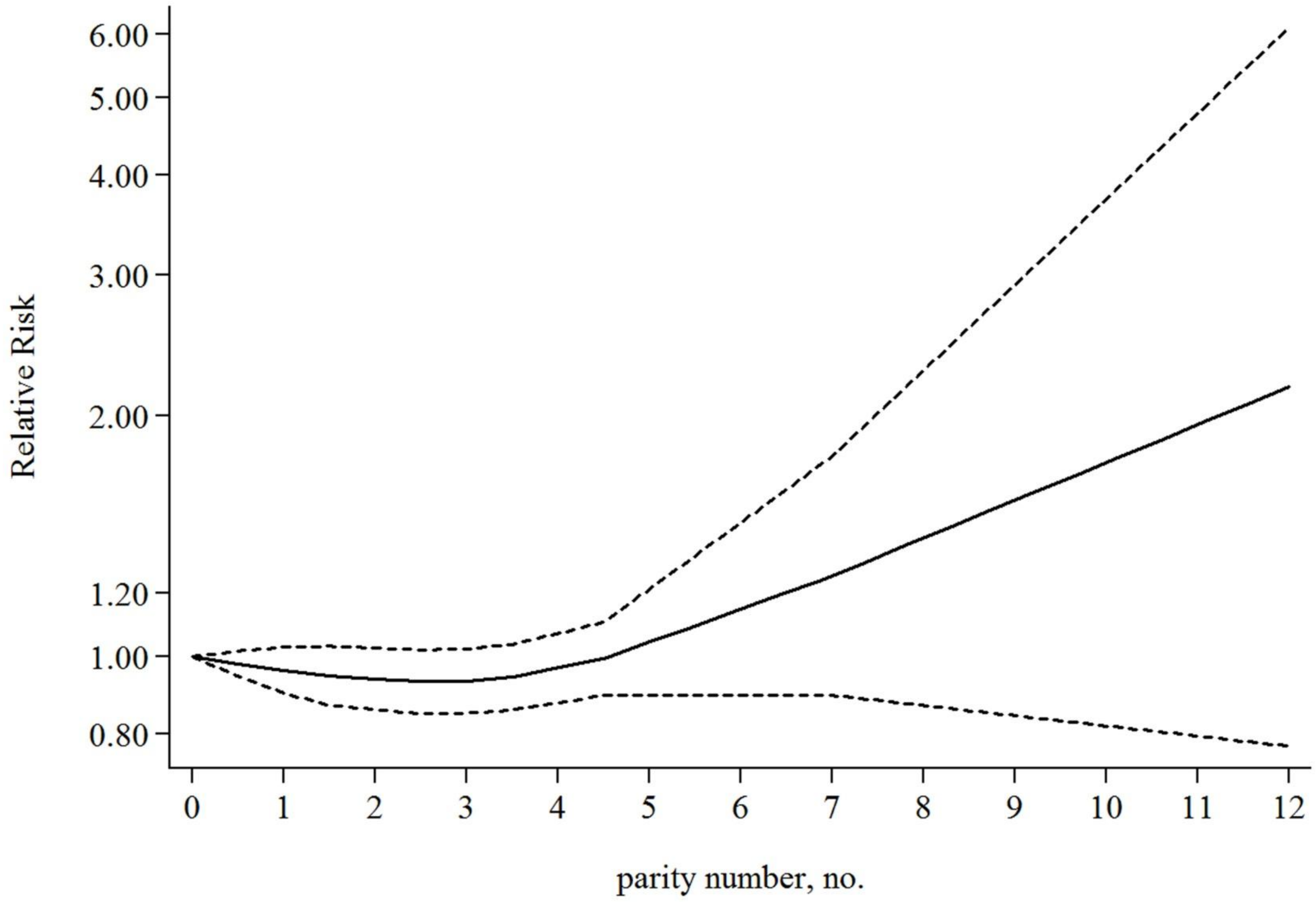

6.00
5.00
4.00
3.00
2.00
1.20
1.00
0.80
Relative Risk
0
1
2
3
4
5
6
7
8
9
10
11
12
parity number, no.

**Supplementary Figure S1.** Dose-response relationship for the association between parity number and gastric cancer risk. The solid line represents the estimated relationship. The dashed line represents the 95% confidence interval of the estimated relationship. The P values for nonlinearity were 0.093. The vertical axis is on a log scale.
